# Supplementary material for: The potential of electricity transmission corridors in forested areas as bumblebee habitat
Source: R Soc Open Sci. 2016 Nov 23;3(11):160525. doi: 10.1098/rsos.160525 (PMC5180138; doi:10.1098/rsos.160525)
Supplement: Supplementary Material for Hill & Bartomeus: The potential of electricity transmission corridors in forested areas as bumble bee habitat. [file rsos160525supp1.docx]

Supplementary Material for Hill & Bartomeus: **The potential of electricity transmission corridors in forested areas as bumble bee habitat.**

**Figure S1:** The location of the study area within in the Swedish region of Uppland and the locations of the 10 2km radius landscape areas surveyed. The black outlined areas are bisected by a transmission corridor; the red outlined areas are not (Map: Länsstyrelsen, 2014).


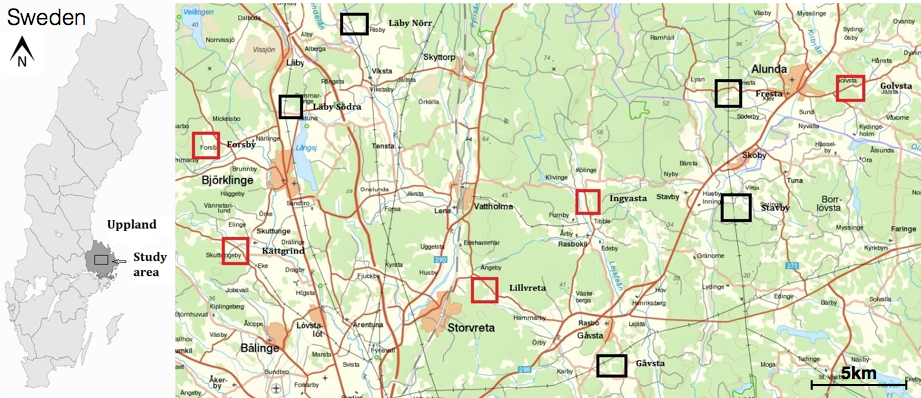


**Figure S2:** Photos representative of the seven habitats surveyed. A) Drains, B) Road margins, C) Grasslands, D) Forests, E)Crop edges, F) Grassland-forest edges and G-I) Transmission corridors.


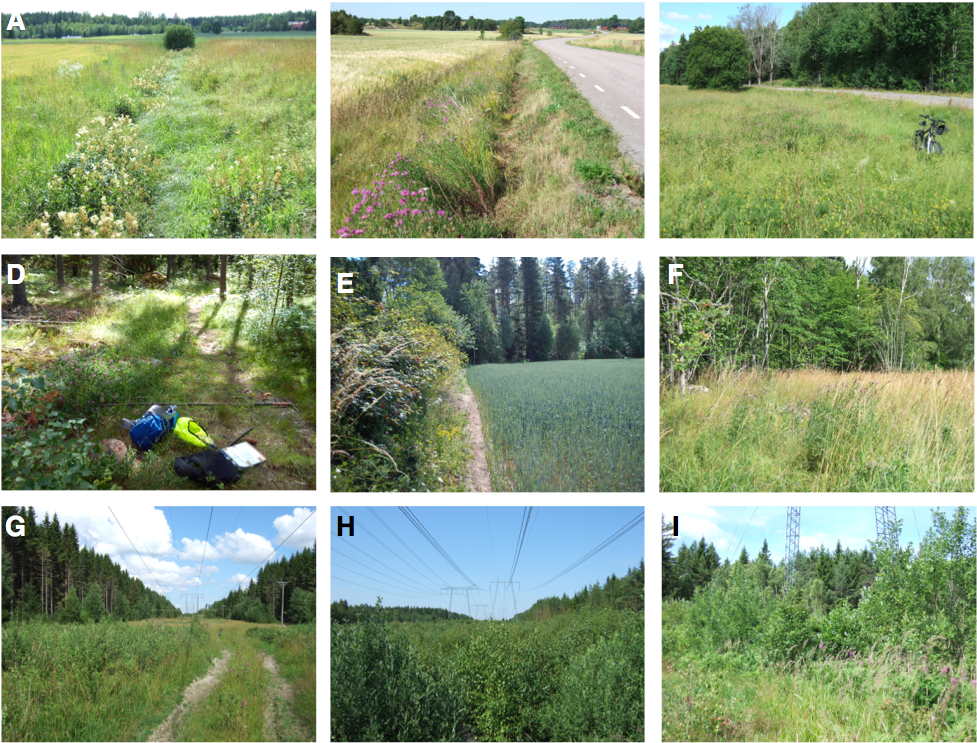


**Figure S3:** Rarefaction curves for each habitat type. The dotted line indicates the sample size used to rarefy gamma diversity per habitat (n = 90 individuals).


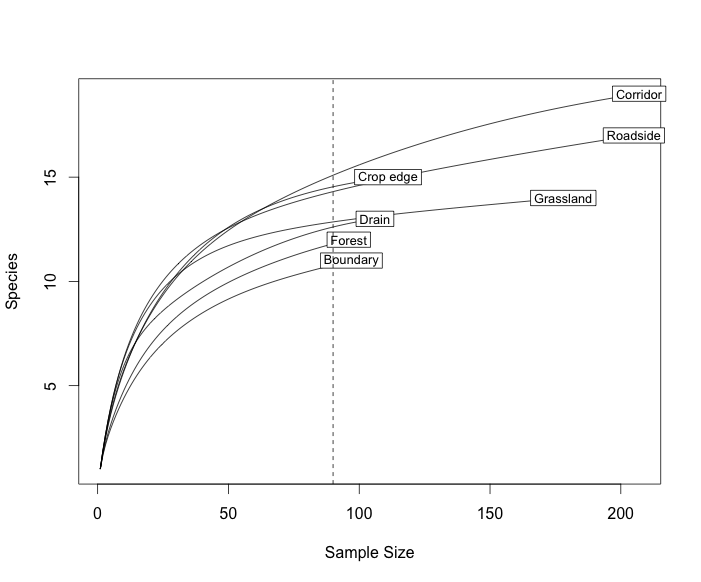


Table S1: Raw data (not corrected by sampling effort) showing the number of individuals of each species collected in each site.

|  | Corridor | Forest | Forest grassland boundary | Maintained ditch | Maintained roadside | Non flowering crop edge | Semi natural grasslands |
| --- | --- | --- | --- | --- | --- | --- | --- |
| *Bombus barbutellus* | 1 | 0 | 0 | 2 | 0 | 4 | 0 |
| *Bombus bohemicus* | 9 | 1 | 0 | 0 | 8 | 0 | 1 |
| *Bombus campestris* | 9 | 1 | 1 | 0 | 1 | 1 | 0 |
| *Bombus hortorum* | 0 | 7 | 10 | 2 | 7 | 4 | 10 |
| *Bombus humilis* | 3 | 0 | 0 | 0 | 1 | 0 | 0 |
| *Bombus hypnorum* | 3 | 2 | 1 | 0 | 2 | 1 | 1 |
| *Bombus lapidarius* | 2 | 0 | 1 | 3 | 10 | 2 | 5 |
| *Bombus muscorum* | 1 | 0 | 0 | 0 | 0 | 0 | 0 |
| *Bombus norvegicus* | 1 | 1 | 0 | 0 | 0 | 0 | 0 |
| *Bombus pascuorum* | 55 | 49 | 54 | 20 | 53 | 42 | 41 |
| *Bombus pratorum* | 11 | 5 | 0 | 0 | 1 | 3 | 3 |
| *Bombus quadricolor* | 10 | 5 | 6 | 1 | 8 | 3 | 7 |
| *Bombus ruderarius* | 2 | 2 | 4 | 12 | 21 | 5 | 10 |
| *Bombus rupestris* | 2 | 0 | 0 | 11 | 4 | 5 | 10 |
| *Bombus soroeensis* | 11 | 3 | 11 | 2 | 11 | 6 | 11 |
| *Bombus spp* | 15 | 11 | 3 | 16 | 22 | 11 | 28 |
| *Bombus subterraneus* | 12 | 0 | 3 | 16 | 15 | 2 | 17 |
| *Bombus sylvarum* | 2 | 0 | 0 | 1 | 10 | 4 | 8 |
| *Bombus sylvestris* | 1 | 0 | 0 | 2 | 1 | 0 | 0 |
| *Bombus terrestris* | 57 | 9 | 3 | 18 | 30 | 18 | 26 |
